# Co-creating community initiatives on physical activity and healthy eating in a low-income neighbourhood in Quito, Ecuador

**DOI:** 10.1186/s41256-025-00412-2

**Published:** 2025-04-17

**Authors:** Sergio Morales-Garzón, Elisa Chilet-Rosell, María Hernández-Enríquez, Francisco Barrera-Guarderas, Ikram Benazizi-Dahbi, Marta Puig-García, Andrés Peralta, Ana Lucía Torres-Castillo, Lucy Anne Parker

**Affiliations:** 1 Present Address: Department of Public Health, History of Science and Gynaecology Department, Miguel Hernández University, 03550 Alicante, Spain; 2 CIBER in Epidemiology and Public Health, 28029 Madrid, Spain; 3 Faculty of Medicine, Pontificia Universidad Católica del Ecuador, Quito, Ecuador; 4 Institute of Public Health, Pontificia Universidad Católica del Ecuador (PUCE), 170143 Quito, Ecuador

**Keywords:** Co-creation, Physical activity and healthy eating, Non-communicable diseases

## Abstract

Promoting healthy behaviours to improve population health requires strategies that foster supportive environments and actively engage communities. Here, we detail an evidence-informed co-creation process in Ferroviaria, a low-income neighbourhood in Quito, Ecuador, led by six members of a local Women’s Association. Co-creation here involved a participatory approach that integrated local epidemiological data on non-communicable disease (NCD) risk factors and employed the "Dialogue Forum" methodology to guide collective decision-making. Through this process, community leaders and participants prioritized initiatives based on community needs, ultimately choosing to develop a community food garden. This initiative fostered social confidence, empowerment, and cohesion, though challenges emerged, including limited participation in the evaluation phase and difficulties in sustaining actions post-implementation. We demonstrate how the "Dialogue Forum" can be an effective tool for inclusive decision-making in low-resource settings. For future co-creation efforts, we underscore the importance of building strong local networks and implementing strategies to prevent dominance by either community leaders or researchers within the co-creation process. Initiatives should incorporate comprehensive community-led evaluations, and it is critical for community leaders to understand the importance of developing actions that the community can independently lead and sustain without continued institutional support. When health data is used, it should be framed to emphasize contextual determinants rather than focusing solely on behavioural risk factors. This report contributes to the growing evidence on community-driven health interventions, positioning co-creation as a pathway to social change and enhanced public health outcomes.

**Supplementary Information:**

The online version contains supplementary material available at 10.1186/s41256-025-00412-2.

## Introduction

According to the Pan American Health Organization [1], 81% of total deaths in the Americas in 2019 can be attributed to non-communicable diseases (NCDs), and 39% of those deaths were among people under 70 years old. Promoting physical activity and healthy eating at a population level can help avert these conditions [2]. In Ecuador, where this participatory initiative was conducted, NCDs were responsible for the loss of more than 3 million disability-adjusted life-years (DALYs) in 2021 [3]. Findings from the World Health Organization (WHO) STEPwise NCD risk factor surveillance survey conducted in Ecuador in 2018 [4], highlightedhealthy eating and phisical activity as the main challenges faced by the country, whilst recognising that behaviours that are highly influenced by the social environment and the economic situation of the population. The survey showed that 94.6% of adults consume less than 5 portions of fruit and vegetables per day as recommended by WHO, and overweight and obesity affect more than 75% of Ecuadorians with a higher prevalence in women [4]. It also highlighted higher salt and sugar intake than recommended, and the rate of processed food consumption was high among people aged 18–44. Low socioeconomic status, particularly in low-income settings, is associated with unhealthy dietary patterns, health inequalities in terms of access to health services, and an increased risk of NCDs [5]. This issue directly affects Ecuador, where socioeconomic data from 2021 showed that poverty affected more than 30% of the population [5]. Therefore, an evidence-informed co-creation process was propsed in this perspective to support decision-making for healthy eating and phisical activity.

## Including the community in health promotion

Although NCDs can be prevented through behavioural changes, individual behaviour is influenced by many factors, including the circumstances in which people live and work [6]. This underlines the importance of developing strategies, tools, and actions to improve community health by creating environments that promote behaviours that are positive for health [7]. A fundamental part of public health work is engaging communities in health promotion, which recognises health as a collective goal requiring collective efforts [7]. NCDs are closely linked with protective behaviours such as physical activity and healthy eating, which can be promoted with community actions that create social capacity and empower communities to decide and implement initiatives to address their health concerns [8–10].

Communities, defined as heterogeneous networks of people, institutions and associations joined by an element of union, such as geographical or identity factors, are fundamental in promoting behavioural changes because collective actions can motivate individual changes [11]. In this case, we use the term community to refer to the people who live in a low-income neighbourhood in Quito, Ferroviaria. To engage communities in public health initiatives, various theoretical frameworks, such as Community-Based Participatory Research or Participatory Action Research, prioritise community participation and implication, empowering participants to play an active role throughout the research process [12]. One such framework, is the “Dialogue Forum”, developed by the European Union and designed to connect participants and policymakers in a common decision-making process [13]. We used this framework because it incorporates participatory dynamics. Including communities in public health design has been shown to promote social change and community empowerment [14], which is an effective practice for addressing health issues in public health [12, 15–18].

While participatory research, including co-creation processes, is grounded in the essential role of community participation in the decision-making process [19], it also presents limitations. There is a significant criticism of this type of practice, arguing that conducting exclusively participatory health promotion research is severely restricted due to the general mismatch between the capacities, implications and objectives of participants, stakeholders, and academics [20, 21]. Given the absence of similar research, we emphasise the need to publish scientific experiences of public health initiatives using participatory frameworks in low-income settings [12]. Here, we decided to co-create a health promotion action, advocating that community participation in health must be understood from its capacity to address and create tailored strategies that can improve the health of the community within the community itself. We describe a context-specific and evidence-informed co-creation process to promote physical activity and foster healthy eating habits within a low-income neighbourhood in Quito, Ecuador. We share the challenges we encountered and propose recommendations for future global health practice.

## Co-creating process: a community initiative to promote health

We describe a collaborative process involving researchers, community leaders, and residents working together to co-create a community initiative using a standardised framework. In this project, participants discussed data extracted from a population survey on NCDs risk factors previously conducted by the CEAD project in their context. To facilitate the discussion, the data was transformed into user-friendly infographics. The participants had time to discuss and explain the potential reasons behind the frequency of the different risk factors according to their perceptions and how they could tackle them within the community. Subsequently, participants proposed community initiatives, and after prioritisation, a community action was implemented and evaluated collaboratively.

The co-creation process used involved 5 phases (Table 1), and we can classify the participants into three main groups: (1) the academic team, which included researchers from University Miguel Hernández (UMH) and Pontificia Universidad Católica del Ecuador (PUCE), were responsible for the formal aspects of the workshops; (2) the community leaders, who were responsible for coordinating the process, facilitating workshops, and engaging participants; and (3) the neighbourhood residents, who participated actively in the process or the action’s implementation.

**Table 1:** Overview of the co-creation process

Phase	Participants	Objectives
(I) Community identification	Academic team	To discuss the method and select the neighbourhood where the activity should take place. To discuss and assign roles to team members.
(II) Engagement of community leaders	Academic team and community leaders	To present the proposed research methods to leaders, propose their participation, discuss feasibility and practical issues regarding participant recruitment. To conduct a training workshop using the "Dialogue Forum".
(III) Co-creation workshop	Academic team, community leaders and neighbourhood residents	To discuss and reflect on the data from our STEPS survey, propose individual and collective action using the “Dialogue Forum” and finally select one action by voting.
(IV) Implementation	Academic team, community leaders and neighbourhood residents	To put the selected action into practice.
(V) Evaluation	Academic team, community leaders and neighbourhood residents	To evaluate the methodology used during the co-creation process, as well as the implementation of the action, and its evolution.

We recognize that collaborative processes, like this one, often involve inherent power imbalances, particularly between academic team and community members [22]. In this project, these dynamics were further shaped by the involvement of researchers from a high-income country and the European-funded CEAD project. To mitigate these imbalances, we intentionally prioritized local leadership by ensuring that one of the main academic facilitators was from PUCE and had extensive experience and training in community development. Through this approach, we wished to strengthen trust, ensure cultural and contextual relevance, and foster an environment where community voices were central to decision-making.

### Phase (I) Community identification

The process outlined is part of the Contextualising Evidence for Action in Diabetes in Low-resource Settings (CEAD) project, led by UMH in collaboration with the PUCE and the Centre for Community Epidemiology and Tropical Medicine (CECOMET). The initial research proposal was based on the premise that, despite ample evidence highlighting the importance of promoting physical activity and healthy diets to prevent diabetes and other NCDs, significant barriers to implementation persist, especially in low-resource settings. As part of the CEAD project, we conducted community dissemination events to share and discuss the findings from a population survey on NCD risk factors (A Spanish language report on this exercise can be found here: https://zenodo.org/records/10682319). During this process we identified the Ferroviaria neighbourhood as a potential site for this co-creation activity. The selection was based on the low level of health community organisation that researchers observed in these events and perceived social exclusion. This neighbourhood, located inside 17D06 health district, has a population of 120,000 inhabitants and was identified in the 2022 Quality of Life Report as one of the most poverty and insecurity-affected areas in the Eloy Alfaro district of Quito. This diverse yet predominantly low-income community faces significant exclusion, given primarily by poverty and limited access to basic health services [23].

### Phase (II) Engagement of community leaders

After selecting the potential neighbourhood, the academic team reached out to a Women’s Association that operates within the area, with its community leaders being residents. Six leaders from this association expressed keen interest and were invited to an initial community meeting held at the Women’s Association headquarters on 11 May 2022. The meeting focused on learning about their previous experience in community work. The leaders explained that the neighbourhood was divided into three sectors and highlighted difficulties in working simultaneously in all three sectors due to political rivalries which affected the social organisation between sectors. After discussion, we concluded that the best approach was to focus on High Ferroviaria, given the need for and absence of community initiatives in this area. During this phase, we also held a training workshop on the 18th of May 2022 which involved a dialogue forum framework decision process. The research team was the facilitators and the community leaders as the participants. With a group discussion of the methods, we also sought to emphasise the leadership role of the community leaders in the subsequent cocreation workshop. This workshop lasted 2 h and 30 min.

### Phase (III) Cocreation workshop

With support from the academic team, the community leaders organised the recruitment of neighbourhood participants and scheduled a co-creation workshop on 21 May 2022. Twenty residents participated, seventeen of whom were from an elderly adults' association, '60 y Piquito,' and three from a youth association, 'Jóvenes de la Ferroviaria.' Most participants were women (N = 15, 75%). The workshop followed the “Dialogue Forum” steps to prioritise and develop a collective decision-making process aimed at creating an inclusive space to generate actions and initiatives through discussion and participation.

**Fig. 1:**
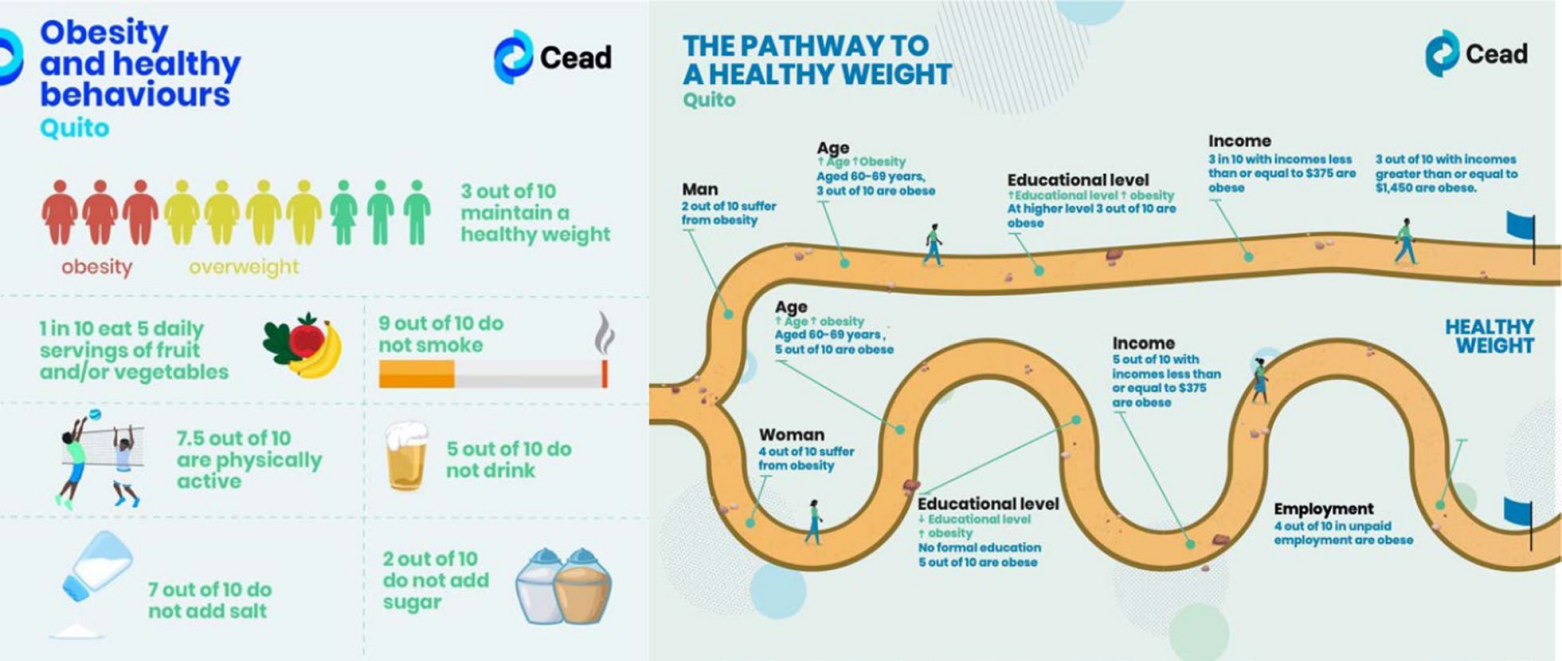
Infographics presented in the co-creation workshop displaying some of the findings from a local survey on behavioural NCD risk factors, and how sociodemographic characteristics influence obesity

Workshops began with presenting and discussing data on NCD risk factors collected through a population survey carried out in the same district during 2020–2021. This data was collected from a STEPwise survey for NCD risk factor surveillance [24] conducted by the CEAD project. As mentioned above, data was presented in two simplified infographics (Fig. 1), [Spanish Versions can be found in the online supplement Figure S1]. Participants were encouraged to reflect on the data based on their own anecdotal or narrative relationship to the survey findings. After discussing the data and considering the project's focus on health promotion, moderators introduced the key question: “*Given that we know physical activity and healthy eating help us live better, what can we do as a community to promote them?*” to induce reflection among the participants and facilitate the following steps involved in the workshop.

The participants were divided into three working groups, each led by two moderators from the leader’s community group. The aim was to discuss and identify actions in physical activity or healthy eating that could be transformed from individual actions into collective initiatives. Each participant presented an idea, which was then discussed by the group and transformed into a collective initiative. For example, if the individual initiative was 'I should eat more fruits and vegetables,' the collective initiative could be transformed into 'we can organise a workshop on new ways of cooking with fruits and vegetables'. After discussing each collective action, the groups identified the most achievable collective actions, describing the fundamental obstacles and opportunities associated with each one. In the case of the example above, a barrier might be how we can ensure that these workshops present healthier culinary options, and the opportunity might be to ask the university to provide nutritional support for the workshop. This step was crucial as it allowed participants to select and prioritize actions based on their feasibility considering the community’s resources. If an idea was beyond the community's control, this step addressed it. For example, prohibiting ultra-processed foods in local groceries would not be feasible within the community’s capacity. Finally, each group selected one action and defined central aspects such as the location, schedule, and necessary resources. They presented their proposed action to the entire group of workshop participants and voted by show of hands for the most achievable action. The first group proposed to create a legal association to promote health actions, particularly in physical activity and healthy eating. The second group proposed a community food garden, incorporating workshops and educational activities to promote gardening and healthier eating. The third group proposed a healthy recipe book based on traditional knowledge and healthy eating. The community food garden received most votes, and everyone agreed on this majority decision. They suggested transforming the backyard of the Women’s Centre into the garden, with plans to begin on 27 May 2022. This workshop lasted 3 h.

### Phase (IV) Implementation

After defining the core elements of the selected action, community leaders invited additional members to participate in its implementation. The academic team supported this by providing materials not readily available within the community. The food garden was implemented through a “Minga”, a communal practice in Ecuador and other Latin American countries, where the community selects a day for voluntary community work. Twenty-eight residents participated in transforming of the backyard of the Women’s Centre into a food garden. One of the leaders contacted the Plurinational and Intercultural Conference on Food Sovereignty (Span. COPISA), a governmental institution that advised on the transformation process. The academic team provided fertile soil for gardening, vegetable seeds, and germinated seeds. This event lasted 3 h, and the community was heavily involved in the process. They also proposed installing a “Cui” (guinea pig) nursery next to the garden with support from the COPISA collaboration.

### Phase (V) Evaluation

We evaluated the decision-making process, perceived effectiveness, and sustainability of the initiative using two interviewer-administered questionnaires. Each question was rated according to a scale from 0 to 5, [S.2]. All participants involved in the process and implementation were invited to an initial evaluation immediately after the action. A second evaluation, with the same scale and a series of open questions [S.3], took place six months later during a meeting for the food garden’s maintenance. Participation in the evaluation was voluntary, and not all members took part. We also assessed the perceived health impact effectiveness of the action and considered its potential role in fostering community cohesion.

Nine people participated in the first phase of the evaluation (seven residents and two community leaders, with eight having taken part in the “Minga”), and 12 participated in the second evaluation six months later (ten residents and two community leaders). Overall, participants viewed the decision-making process positively in terms of participation, simplicity, and the appropriateness of the methodology. However, half of the participants felt that the follow-up and support from the research team after the action implementation were inadequate. Participants indicated a positive impact of the action on knowledge of healthy habits, but this general sentiment changed after six months, as they reported no improvements in healthy habits and described the outcome as unrealistic or unaffordable for the community. Participants indicated in the initial questionnaire an improvement in community unity because of the action. However, the six-month evaluation contrasted this information as they reported a decline in community commitment and observed no growth in the action. Some participants indicated that there was a lack of community representativeness. The community reported that they had previously experienced a failed garden. Additionally, despite plans to improve the garden by creating a nursery with COPISA support, they encountered sustainability issues and saw no progress towards their objective (Additional file 1).

## Achievements and challenges of the co-creation method

We describe our experience of using local data on the prevalence of NCD risk factors to empower community members and co-design context-specific and evidence-informed actions to promote phisical activity and fostering healthy habits in Quito, Ecuador. The implementation of the Dialogue Forum framework, as described by Aguirre et al. [25], helped community members identify action-oriented approaches, ensuring that all perspectives presented included a practical component rather than solely theoretical ideas or propositions without empirical grounding. This framework has been recommended for integrating experiences and various forms of knowledge to create community-led actions [23]. Our initial proposal focused on physical activity and healthy eating which may seem to be prescriptive and top-down at first as the priorities were not immediately identified by the community. As mentioned previously, the initiative was part of the CEAD project which focussed on diabetes prevention in low resource settings and sought to generate information on how research data and participatory initiatives can be used to promote physical activity and health eating. However, the community suggested actions that extended beyond the scope of our initial proposal, such as the creation of a working group advocating for an association to facilitate health initiatives or the creation of a guineapig nursery. This development highlights the importance of recognizing and fostering the community's capacity to identify and address health issues that are most relevant to their needs and priorities. According to Popay et al., empowering disadvantaged communities and promoting community capacity requires achieving structural transformations that go beyond an inward gaze, such as just promoting individual changes in healthy eating within community, and are motivated by the collective sense of control over the initiative [26].

As introduced earlier, the success of participatory approaches should be evaluated not only by their ability to produce quantifiable health benefits but also by their capacity to generate social empowerment and instil confidence within the community to drive actions. This shift acknowledges that the process of engaging community members in the identification, planning, and implementation of health initiatives is as valuable as the outcomes themselves [27]. Although this experience demonstrated limited sustainability, we reaffirm findings from previous studies that by creating spaces for dialogue, collaboration, and collective decision-making, participatory research can contribute to the development of a more equitable and sustainable approach to health promotion, one that is rooted in the community’s strengths, knowledge, and aspirations [26, 28].

During the evaluation, some of the community members voiced discontent about the decision to establish a food garden, specifically questioning its capacity to promote healthier habits. While the initial prioritization of the community action was determined through an open vote during the workshop, incorporating a deeper group analysis and discussion of each proposal before voting could help ensure broader consensus and alignment with community expectations. The dissatisfaction observed in this initiative, although minimal, could signal a divergence between the proposal's primary objective and the community's perception of its potential outcomes. It is crucial to recognize and address this discrepancy underscoring the importance of aligning research goals with community needs and expectations. Effective participatory research requires a deep understanding of the community's past experiences, current priorities, and aspirations for the future. By actively listening to and incorporating community feedback, researchers can adapt their approaches to better suit the local context and create initiatives that resonate with the community's values and goals. This process of continuous dialogue and adjustment is essential for building trust, fostering ownership, and ultimately enhancing the relevance and sustainability of the intervention.

In this experience, the community positively evaluated the activities conducted. Their perceived improvement in perceived knowledge about healthy habits, the process of creating a food garden from scratch, and developing actions that create community cohesion must be considered achievements. However, participants also reported that their attitudes toward physical activity and healthy eating remained unchanged. This disparity underscores the gap between knowledge acquisition and its ability to motivate behavioural change [29–33]. Here, the initiatives stemming from the food garden creation may suggest that some degree of community cohesion was developed. The leaders expressed interest in creating a nursery for guinea pigs with the collaboration of COPISA, the governmental institution that participated in the “Minga”, and in establishing a juridical association to develop formal actions and negotiate with the local government. These initiatives sparked by the garden creation could be the beginning of the community's growth in confidence and capacity, as evidenced by Zoellner et al. [33], where stakeholder support and the academic team were shown to guarantee social empowerment.

This process of implementing a co-creation framework for the promotion of physical activity and healthy eating in a low-income neighbourhood had several difficulties. Despite their satisfaction with the methodology used, neighbourhood residents perceived that their influence in the decision-making process was lower than expected. This view can be explained by the researcher’s observation of the control and influence of community leaders’ opinions during the co-creation workshop. Similarities between the training exercise performed with community leaders and the co-creation workshop point to this soft power. While community leaders play a positive role in community-based work [34] and are key to well-functioning participatory research, it is important to identify ways to limit their dominance in the decision-making process. In this context, it may be difficult for a community leader to advocate for a position that is not aligned with their own beliefs, potentially serving as a limitation for participants.

During the co-creation workshop, where participants prioritized actions based on the community’s assets, it was crucial to highlight for both the community and its leaders that the process focused on developing actions rooted in community capacity. Some viewed collaborations with the academic team as a chance to seek funding and economic support. Therefore, it was essential for community leaders and community participants to understand that the primary goal was to create actions that the community could lead and sustain. To achieve this, transparency from the outset is critical—particularly regarding the decision-making process, the roles of participants, the scope and reach of the initiative, and the incentives for participation.

In this project, we did not include economic incentives for leaders or participants. While financial compensation has been suggested to address inequalities and promote the inclusion of vulnerable groups [35], it is also important to consider the potential risk of perpetuating inequalities by offering more to leaders in facilitator roles. We recommend a thorough and thoughtful exploration of ways to motivate participation that extend beyond economic incentives, ensuring clarity and transparency about these decisions. Collaboration should be framed as a reciprocal process—where the university gains knowledge and insight, and participants may benefit from the initiative itself, but ultimately, the greatest value lies in the connections built through the process and the potential these relationships hold for future community-led actions. Establishing community contact and identification can be challenging, but difficulties in the initial contact process can be overcome by seeking support from individuals or institutions with prior experience working with the community, such as primary care centres, parishes, churches, and social centres [36]. In our case, the first contact was through a Women's Centre, where women from various community organizations collaborated.

Another limitation was the less-than-optimal evaluation process which resulted in low participation, an aspect that could itself indicate discontent. Initially, we relied on a series of closed questions, which limited the depth and richness of the information gathered. In the second phase, we introduced open-ended questions to elicit more specific feedback, but these were administered by the same individual who led the participatory initiative. This raises the possibility of social desirability bias, where participants’ responses may have been influenced by the presence of the facilitator. We acknowledge the inherent challenges in evaluating co-created health initiatives [37]. For future projects, we recommend integrating evaluation fully into the co-creation process, allowing participants to determine how and when to assess both the process and its outcomes. Open, in-depth exploratory discussion groups could be particularly valuable for capturing nuanced insights [38]. To support this, it may be necessary to provide additional training for participants in qualitative and evaluation methodologies, empowering them to lead these processes effectively.

Regarding participation, six of the nine participants in the evaluation were from one of the three social organisations mentioned earlier: a women’s group, an elderly group, or a youth group. Their involvement may have been motivated by their experience with group activities. Participants viewed the sustainability of the action as limited, citing a lack of institutional support and follow-up. As highlighted by Attree et al., engaging communities in research projects presents significant challenges, as participation can be influenced by factors like limited time, work commitments, or lack of incentives. [39]. In our case, we were not able to guarantee the continuity of the action once the institutional support ended. Therefore, it is important to consider these potential barriers to participation to keep the community engaged.

Future initiatives should include a better analysis of external factors, such as environmental conditions, that could influence the success or failure of any chosen action. In this case, the decision to create a community garden in a climate with abundant rain could have considered actions or protective structures for sustainability. It is also important to emphasise that community representativeness must be guaranteed. In our case, community engagement lacked the presence of young people and men, which may have contributed to the community's loss of interest and the garden's limited attractiveness to the community, creating a sense of it being exclusively for the women´s group.

Another challenge is the effective use of research data, for which it is essential to ensure that the information presented to the community is understandable and adapted to the public. We adapted and synthesized the data from our population survey with simple sentences and graphic representations of NCD risk factors and their distribution in the health district. However, using epidemiological data focused on risk behaviours at an individual level, such as the WHO STEPS survey used here, may bias the types of solutions proposed towards more individual level interventions. As such, downstream determinants of unhealthy eating and physical inactivity, such as health education and individual choice surrounding what to eat or how to exercise, takes prime position. This has also been recognised by other authors [40, 41] showing that more contextual or political actions that lead to health-promoting environments at the community level take second place. It is also important to remark that, although data presented in the second infographic was designed to spark reflection on health disparities with a gender focus, this aspect was not effectively integrated into the workshop discussion. This may reflect the significance and influence of local culture in shaping community attitudes. What may be perceived as a requirement in one community may not necessarily hold the same value in another [42]. Finally, the evaluation process designed here was not useful to explore the impact of the co-creation initiative. We included questionnaires and discussions with participants as part of the initiative to co-create a community health action using our research data, but the need to comprehensively evaluate the participatory process and its role in developing community empowerment was overlooked. All these achievements and challenges are summarized in the Table 2.

**Table 2:** Achievements and challenges found during the research

Achievements	We leveraged local data on NCD risk factors to catalyse community initiatives. The Dialogue Forum proved an effective method for prioritisation and collective decision-making. The co-creation process fostered empowerment and social confidence, leading to innovative extensions beyond the initial proposal, such as partnerships with other groups and developing a guinea pig nursery. The community provided positive feedback on increased knowledge and strengthened cohesion through collective work.
Challenges	Our initial proposal centred on physical activity and healthy eating risks being perceived as prescriptive and not fully aligned with community priorities. We evidenced the community leaders influence inside the co-creation process to prioritise actions, which could shape prioritization toward specific actions and affect balanced representation of broader community interests. Despite knowledge gains, participants indicated limitations in changing attitudes toward physical activity and healthy eating, emphasizing the need for contextually framed solutions over individual interventions. Underrepresentation of certain community groups during the process may have affected the sustainability of the action. We noted low participation in the evaluation and limitations in impact measurement, particularly around community empowerment.

## Implications for global health

For future researchers engaging in this type of participatory action, we recommend several key strategies to foster meaningful community engagement and promote effective interventions. Firstly, establishing community connections is essential, and having a person with networks or contacts within the community can greatly facilitate the development and promotion of participatory actions. In our case, the academic members from PUCE proposed the neighbourhood to work with, and the leaders' group's experience in promoting community actions was crucial for the project's success.

The use of the "Dialogue Forum" method in workshops and selection phases has proven to be an effective way to promote participation in groups and identify actions based on participants' opinions aimed at creating change and social empowerment. By incorporating this method into our co-creation process, we show how it can provide communities with the autonomy to transform research data into social change. However, it is essential to recognize that this social change should be driven by the community and strategies are needed to prevent group leaders or researchers from dominating in the decision-making process. If research data is to be used to trigger action, it is important to consider how it is framed. Attention is needed to ensure that the data presented recognises that individual behaviours are ultimately framed by contextual determinants of health. It is also important to state that there are reports which emphasize the importance of including mechanisms to ensure gender parity and inclusivity in participation, which can improve the quality and effectiveness of participatory projects [43]. Addressing power dynamics within the process is essential for effective community empowerment, which does not merely consist of achieving individual changes within a group. True empowerment involves transforming structural models of control where collective control capabilities serve as forms of positive power for the community [26].

The evaluation process should be integrated into the co-creation phase, with community members actively involved in determining how and when the evaluation takes place. In this process, the academic team should adopt a facilitative role, sharing knowledge of evaluation approaches that include both quantitative and qualitative methods. To comprehensively assess the project's impact, we recommend designing an overarching evaluation that includes both pre- and post-intervention data collection that goes beyond measuring the perceived impact of the initiative at meeting its goal. Measures of community cohesion and social capital could be useful to this end.

## Supplementary Information

Additional file 1.

## Data Availability

All data generated or analysed during this study are included in this published article and its supplementary information files.

## Abbreviations

NCDs: Noncommunicable diseases
UMH: Universidad Miguel Hernández de Elche
PUCE: Pontificia Universidad Católica de Ecuador
