# Supplementary material for: Co-creating community initiatives on physical activity and healthy eating in a low-income neighbourhood in Quito, Ecuador
Source: Glob Health Res Policy. 2025 Apr 17;10:18. doi: 10.1186/s41256-025-00412-2 (PMC12004727; doi:10.1186/s41256-025-00412-2)
Supplement: Supplementary file 1 — Additional file 1. [file 41256_2025_412_MOESM1_ESM.docx]

**Supplementary material**

**S1:**  Infographics presented in the co-creation workshop displaying some of the findings from a local survey on behavioural NCD risk factors, and how sociodemographic characteristics influence obesity, Spanish version.


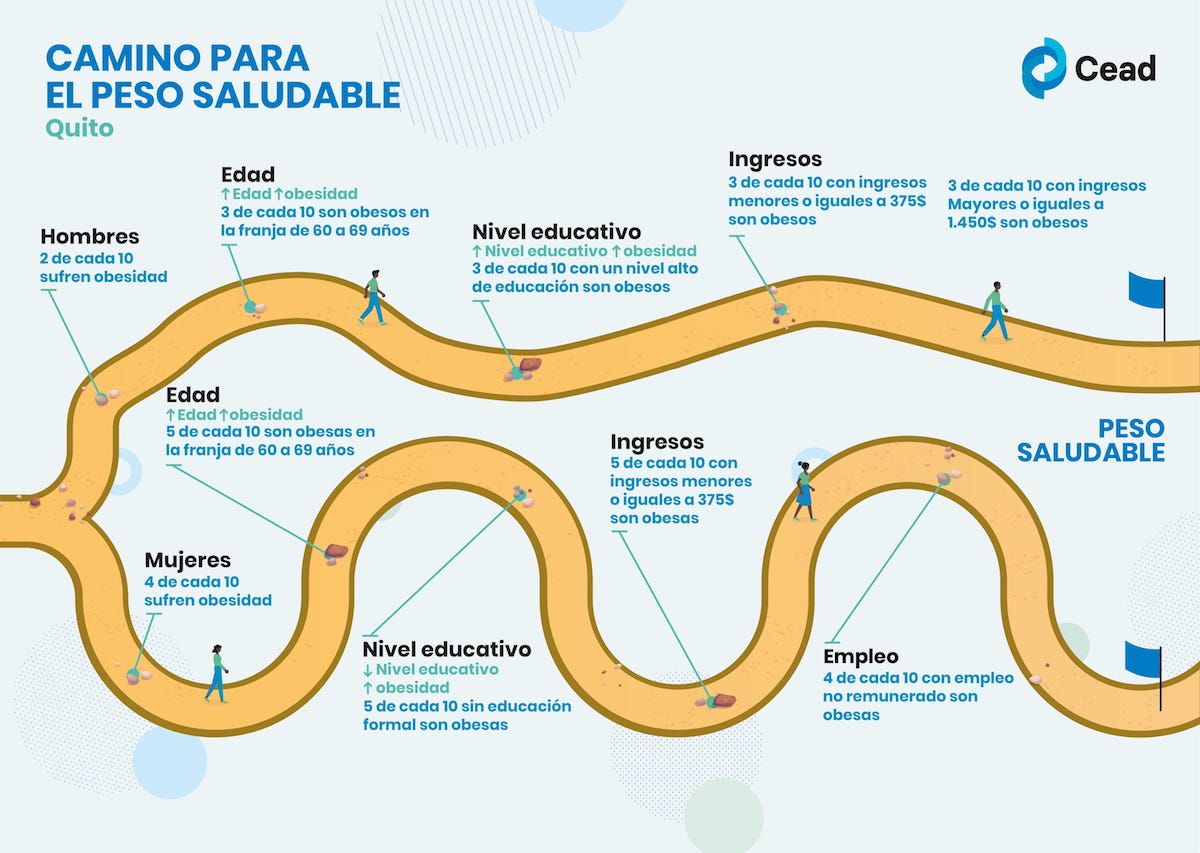

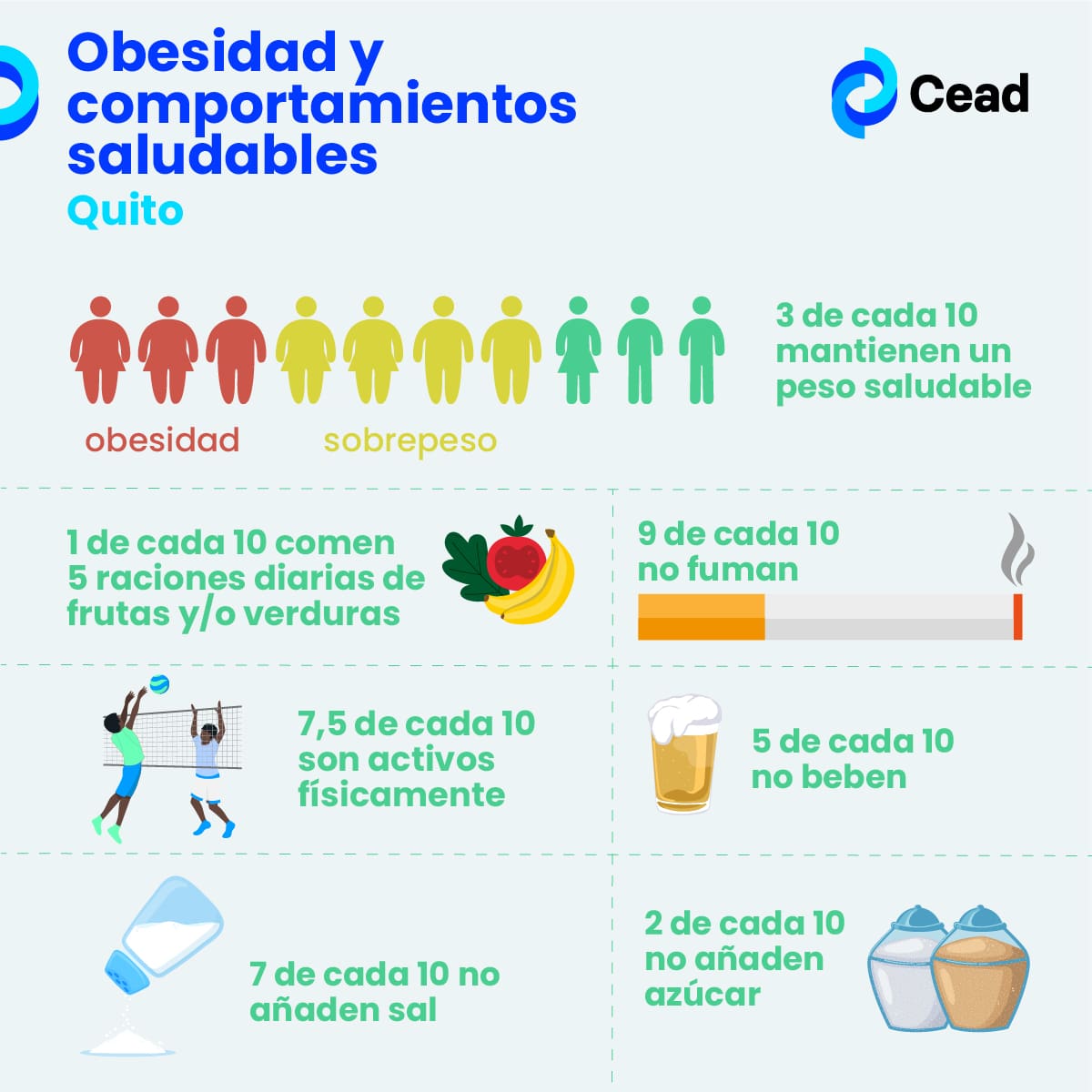


| INITIAL EVALUATION | Question included in questionnaires  **S2**: Questions included in initial and six-month evaluation. | Translation used in questionnaires |
| --- | --- | --- |
|  | 1. Do you consider your participation in the workshop to have been productive? | 1. ¿Consideras que tu participación en el taller ha sido productiva? |
|  | 2. Do you think the process was community-led? | 2. ¿Crees que el proceso fue liderado por la comunidad? |
|  | 3. Do you think that the methodology used is suitable for working with the community? | 3. ¿Consideras que la metodología utilizada es adecuada para trabajar con la comunidad? |
|  | 4. Do you think the identification procedure was straightforward? | 4. ¿Crees que el procedimiento de identificación fue sencillo? |
|  | 5. Do you think the decision was realistic for the community's capacity to act? | 5. ¿Consideras que la decisión fue realista en función de la capacidad de actuación de la comunidad? |
|  | 6. Would you use the methodology if you had to organise a group decision making process? | 6. ¿Utilizarías la metodología si tuvieras que organizar un proceso de toma de decisiones grupal? |
|  | 7. Do you think the procedure could be improved? | 7. ¿Crees que el procedimiento podría mejorarse? |
|  | 8. Did you participate in the community action decided at the workshop? | 8. ¿Participaste en la acción comunitaria decidida en el taller? |
|  | 9. How do you feel in terms of satisfaction with the action taken? | 9. ¿Cómo te sientes en términos de satisfacción con la acción tomada? |
|  | 10. Do you think the action has improved your healthy habits? | 10. ¿Crees que la acción ha mejorado tus hábitos saludables? |
|  | 11. Do you think the action will bring about an improvement in community togetherness? | 11. ¿Crees que la acción contribuirá a mejorar la cohesión comunitaria? |
|  | 12. Do you think it is a sustainable project? | 12. ¿Consideras que es un proyecto sostenible? |
|  | 13. Do you think that the implementation of the action could be improved? | 13. ¿Crees que la implementación de la acción podría mejorarse? |
| SIX-MONTH EVALUATION | 1. Do you consider that the action taken has been effective in improving Physical Activity and Healthy Diet? | 1. ¿Consideras que la acción tomada ha sido efectiva para mejorar la Actividad Física y la Dieta Saludable? |
|  | 2. Do you consider that it has been an action that has grown and improved since its implementation? | 2. ¿Consideras que ha sido una acción que ha crecido y mejorado desde su implementación? |
|  | 3. Do you think that the action has met the objectives of the community at the beginning of the action? | 3. ¿Crees que la acción ha cumplido con los objetivos de la comunidad al inicio de la acción? |
|  | 4. Do you consider the follow-up and support provided by the research team after the action to be adequate? | 4. ¿Consideras adecuado el seguimiento y apoyo proporcionado por el equipo de investigación después de la acción? |
|  | 5. Do you think the action has improved your healthy habits? | 5. ¿Crees que la acción ha mejorado tus hábitos saludables? |
|  | 6. Do you think your fruit and vegetable intake has increased since the action took place? | 6. ¿Crees que tu consumo de frutas y verduras ha aumentado desde que se implementó la acción? |
|  | 7. Do you think that after doing the action you take more care of your diet? | 7. ¿Crees que después de hacer la acción cuidas más tu dieta? |
|  | 8. Do you think the decision was realistic for the community's capacity to act? | 8. ¿Consideras que la decisión fue realista para la capacidad de actuación de la comunidad? |
|  | 9. Did the community maintain their commitment to the action undertaken? | 9. ¿La comunidad mantuvo su compromiso con la acción emprendida? |
|  | 10. Do you think it is an effective methodology for group decision-making? | 10. ¿Crees que es una metodología efectiva para la toma de decisiones en grupo? |
|  | 11. Do you think it is a sustainable project? | 11. ¿Consideras que es un proyecto sostenible? |
|  | 12. Comments, proposals for improvement, limitations of the garden and other core elements | 12. Comentarios, propuestas de mejora, limitaciones del jardín y otros factores clave |

**S3**: Comments provided by participants in the open questions of the six-month evaluation.

| Role | Sex | Literal meaning | Translation |
| --- | --- | --- | --- |
| Leader 1 | Female | 1. Faltó adecuar mejor la tierra, prepararla y abonar 2. Faltó como enamorar al grupo sobre el proyecto | 1. There was a lack in the soil adaptation, the preparation and its fertilisation 2. It lacked how to make the group fall in love with the project. |
| Neighbourhood resident 1 | Female | 1. Porque mejora la salud 2. Luego de tanta lluvia no   seguimos con la acción   1. Faltó educación | 1. It improves health 2. After so much rain, we didn't continue with the action 3. There was a lack of education |
| Neighbourhood resident 2 | Female | 1. Mucho aguacero y yo no puedo trabajar en la lluvia, faltó aflojar bien la tierra y no se hizo 2. Hay muchos adultos mayores, pocos jóvenes que pueden trabajar mejor, hacer macetas y tanques 3. Sí hay seguimiento y más trabajo | 1. It was raining heavily, and I can't work in the rain, the soil needed to be fertilised and it was not done 2. There were many older adults, few young people who can do a better work, make pots and tanks 3. Yes, there were follow-up and more work |
| Leader 2 | Female | 1. El inicio si cumplió, pero luego se perdió 2. Al inicio sí hubo seguimiento, luego que se perdió por el granizo. 3. Porque aprendimos que es necesario consumir alimentos saludables 4. Porque al rato hubo ilusión y después no se trabajó suficiente 5. Si se puede hacer un proyecto sostenible si hay entusiasmo 6. Solo se cosecharon rábanos y no supimos plantar más | 1. At the beginning it was follow-up, but then, it was lost 2. At the beginning there was tracking, but then it was lost due to the hail 3. We have learned why it is necessary to eat healthy food 4. At the beginning, there was enthusiasm, then our work was not enough 5. It is possible to make a sustainable project if there is enthusiasm 6. Only radishes and lettuce were harvested, and we couldn't learn to eat other vegetables |
| Leader 3 |  | 1. No ha crecido porque no tenemos costumbre de siembra, falta de cuidado y mal temporal, se cubrió to de hielo 2. Al inicio de la actividad sí porque participaron todos los integrantes del grupo 3. El inicio fue adecuado el seguimiento, pero luego del mal temporal ya no hubo seguimiento 4. En la alimentación he mejorado porque aprendí a consumir productos más sanos como verduras que poco consumía 5. Sí, ha mejorado la alimentación 6. Fue un sueño que no se pudo realizar, porque se descuidó | 1. It hasnt grow because we are not used toplanting, because lack of care and bad weather, everything was covered by hail 2. At the beginning of the activity all the group members´ participated. 3. At the beginning the follow-up was adequate, after the bad weather there was no follow-up. 4. My diet improved because I have learned to eat healthier products, such as vegetables which I used to eat very little of. 5. Yes, diet has improved 6. It was a dream that could not be realise, it was neglected. |
| Neighbourhood resident 3 | Female | 1. Al inicio, pero luego ya no hubo seguimiento 2. Porque antes comía mucho arroz 3. Es sostenible pero no nos dedicamos | 1. At the beginning, but then there was no more follow up 2. I used to eat a lot of rice 3. It was sustainable but we did not do it |
| Neighbourhood resident 4 | Female | 1. Mejora la salud física por el ejercicio de sembrar y también hace bien estar saludable 2. Porque el temporal no ayudó 3. Al inicio si cumplió, luego vino el granizo y el temporal que mató el cultivo 4. El seguimiento fue al inicio, pero luego del grupo y del tiempo ya no se dio 5. No se dio ninguna cosecha 6. Sí consumo más frutas y verduras que compro 7. Si ayudara el temporal, sí es posible 8. Nos dio pena que no se podía hacer mucho por la gran cantidad de lluvia y granizo todos los días 9. La metodología es buena | 1. It improves physical health through the exercise of sowing and it is also good to be healthy 2. The storm did not help 3. At the beginning it did, but then, came the hail and the storm that killed the crop 4. The follow-up was at the beginning, but after that, the group and time no longer occurred 5. There was no crop 6. Yes, I consumed more fruit and vegetables 7. If the storm would help, yes it is possible 8. We were sorry, that not much could be donde because of the heavy rain and hail every day 9. The methodology is good |
| Neighbourhood resident 5 | Female | 1. El temporal y la falta de seguimiento influyó | 1. The storm and the lack of follow-up had an influence |
| Leader 4 | Female | 1. Cosecharon rábanos y lechugas 2. Faltó dedicación, perseverancia, especialmente después del aguacero 3. Sí es sostenible pero las personas no tienen las herramientas necesarias | 1. It was harvested radishes and lettuces 2. There was a lack of dedication, perseverance, especially after the storm 3. Yes, it is sustainable but people do not have the necessary tools |
| Neighbourhood resident 6 | Female | 1. Cayó granizo 2. Especialmente para trabajar en la casa en macetas | 1. Hail fell 2. Especially for working in the house in flower pots |
